# Commentary: Chemiexcitation of melanin derivatives induces DNA photoproducts long after UV exposure

**DOI:** 10.3389/fphys.2015.00276

**Published:** 2015-10-06

**Authors:** Rajendra P. Gajula, Shobhan Gaddameedhi

**Affiliations:** Experimental and Systems Pharmacology, College of Pharmacy, Washington State UniversitySpokane, WA, USA

**Keywords:** dark CPDs, melanin, melanoma, oxidative DNA damage, UV radiation

Cutaneous malignant melanoma (CMM) is one of the most rapidly increasing cancers in the western world (Bowden, 2004). Frequency of its occurrence is closely associated with skin color and depends on geographical zone (Leiter and Garbe, 2008). Multiple epidemiological studies in humans (Whiteman et al., 2007) and experimental studies with mouse melanoma models suggest that sunburn during childhood confers the highest risk of melanoma development later in life (Noonan et al., 2001). Ultraviolet radiation (UVR) from sunlight that reaches earth is comprised of 90–99% UVA (320–400 nm) and 1–10% UVB (280–320 nm), as most UVB is absorbed by stratospheric ozone (Bowden, 2004). High energy UVB is strongly absorbed by epidermal genomic DNA resulting in direct DNA damage by generating two major photoproducts: cyclobutane pyrimidine dimers (CPDs) and 6-4 photoproducts (6-4 PPs), both of which are mutagenic and carcinogenic in animal models as well as in humans (Bowden, 2004; Lima-Bessa and Menck, 2005). These photoproducts are solely repaired by the process of nucleotide excision repair (Sancar et al., 2004) and the loss of this repair system is strongly correlated with increased melanoma. This loss results from transformed melanocytes, while non-melanoma skin cancers originate from malignantly transformed keratinocytes (Kraemer, 1997; Bowden, 2004). Although it was established that melanin that is specifically synthesized within melanocytes protects against direct DNA damage by absorbing the energy from UVR (Sinha and Häder, 2002). Recent findings from two independent research groups suggest that melanin might contribute to additional DNA damage and might further lead to increased risk of skin cancers (Noonan et al., 2012; Premi et al., 2015).

Using hepatocyte growth factor/scatter factor (HGF) transgenic mice with a controlled UVR delivering system on neonatal mice, Noonan et al., have unraveled interesting findings regarding UVA and UVB-induced melanomagenesis (Noonan et al., 2012). In their experiments, 3 day old C57BL/6-HGF (Black and melanin pigmented), C57BL/6-c-HGF tyrosinase mutant (Albino and no melanin pigment) transgenic mice were irradiated with both UVA and UVB, then with specific UVA or UVB at a biological dosage. The authors have found that UV induction of melanin contributes to melanomagenesis only by UVA and not by UVB radiation. This UVA-mediated melanoma development was associated with oxidative DNA damage in melanin containing black mice and not in albino mice from the same genetic background. However, the biochemical explanation for melanin associated oxidative DNA damage in pigmented melanocytes needs to be addressed.

In a recent study, Brash and his colleagues have elegantly demonstrated the mechanistic and biochemical pathways of interaction of melanin and its contribution to UV-mediated DNA damage response. In the process, the authors have discovered a novel mechanism of generating “Dark” CPDs (Premi et al., 2015). It is well documented that CPDs are formed within picoseconds after a UV photon is absorbed at thymine or cytosine sites in DNA (Schreier et al., 2007). For the first time, Brash and his colleagues have shown that CPD production continued even several hours after UVA treatment in melanin containing murine melanocytes. These CPDs, generated at a later time after irradiation, were called “Dark CPDs.” However, this phenomena was absent in murine fibroblasts and albino melanocytes which lack melanin pigment. This suggests that melanin might contribute to increased DNA damage through dark CPDs. Also UVB irradiated pigmented melanocytes generated dark CPDs, which are comparable to UVA. Remarkably UVB-mediated CPD production was highest after 4 h of irradiation, while UVA-mediated CPDs peaked at 2 h.

To further examine the effect of UVA on dark CPD generation *in vivo*, the authors have used a transgenic mouse model overexpressing Kit ligand (*K14*-*kitl*), where melanocytes are localized within the epidermis. UVA exposure of these mice showed CPD generation 3 times higher at 2 hours relative to 0 h post-irradiation. The majority of these were cytosine containing CPDs that are responsible for UV-signature C → T mutations as opposed to TT CPDs, which are most typical of cells irradiated with UVR. Further in response to UVA irradiation, melanin generates reactive oxygen species like superoxide's (O${}_{2}^{-\cdot }$). Additionally UVA rapidly induces NADPH oxidase (NOX) and iNOS; the long lasting sources for superoxides (O${}_{2}^{-\cdot }$) and nitric oxides (NO^·^), respectively (Roméro-Graillet et al., 1996; Valencia and Kochevar, 2008). These free radicals cause a peroxinitrite (ONOO^−^) spike (~400 times increase in the flux per hour) that in turn degrades melanin to its degradation products. The melanin degradation products and peroxinitrite were found to pass freely across nuclear membrane of the melanosomes. Therefore, inside the melanosome nucleus the high energy peroxinitrite excites an electron on melanin fragments to form a high energy triplet carbonyl state which has the high energy of a UV photon. This energy is transferred to nascent DNA generating C → T CPDs in the dark, which are prone to be deaminated inducing a C to T transition by tranlesion polymerases (Choi et al., 2006). The sustained generation of dark CPDs is primarily determined by the persistent generation of NOX and iNOS levels by UVA radiation. Hence the study by Brash and his colleagues suggests that melanin might act as a molecular vector by promoting UVR mediated dark CPDs through chemiexcitation and consequently promote UVA mediated melanomagenesis in pigmented mice as previously reported (Noonan et al., 2012).

In conclusion, UVB-melanomagenesis is independent of melanin and induces direct mutagenic DNA photoproducts (Figure 1). In contrast UVA-melanomagenesis is pigment-dependent through oxidative DNA damage where melanin acts as a molecular vector in promoting the disease. The generation of dark CPDs by UVA, a major component of sunlight and tanning beds, suggests that the effects of UVR have been underestimated to date and pose a greater risk overall but are quite avertable. Intervention with superoxide inhibitors like α-tocopherol (Vitamin E) and others like- VAS2870 (NOX inhibitor), amino guanidine (iNOS inhibitor), and Ethyl Sorbate a specific quencher of triplet state were found to completely block dark CPD generation. These inhibitors might be used in sunscreens as preventative measures to protect from direct sunlight as well as from post sun exposure (Premi et al., 2015). Overall these studies show a possible role of melanin in promoting UV-induced melanomagenesis. However, previous studies have shown that melanin plays a protective role in UVR mediated skin cancer in dark skinned individuals with eumelanin by shielding against UV radiation, while pheomelanin is harmful by inducing ROS and DNA strand breaks (Chedekel et al., 1978; Takeuchi et al., 2004; Brenner and Hearing, 2008). This raises the question: Is melanin protective or carcinogenic? Indeed, this still requires thorough investigation.

**Figure 1:**
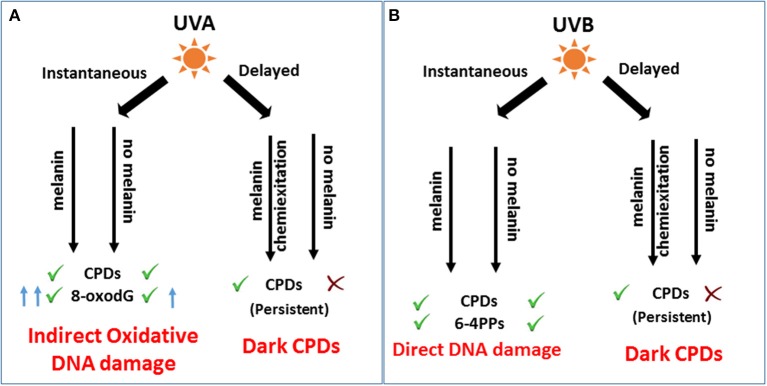
**Role of melanin in immediate and after-effects of UV-DNA damage and melanomagenesis**. **(A)** UVA induces indirect photo-oxidative DNA damage by generating CPDs and 8-oxodG, immediately after irradiation. As a delayed response, UVA induces a significant amount of dark CPDs dependent on melanin pigment by chemiexcitation of melanin fragments leading to melanomagenesis. **(B)** UVB as an immediate response generates mutagenic CPDs and 6-4 PPs to induce direct DNA damage and melanomagenesis independent of melanin. Hours after irradiation, melanin contributes to UVB mediated dark CPDs. However, the role of dark CPDs in melanomagenesis might be insignificant compared to instantaneous CPDs as shown by Noonan et al. (2012).

## Conflict of interest statement

The authors declare that the research was conducted in the absence of any commercial or financial relationships that could be construed as a potential conflict of interest.
